# Supplementary figures and images for: A single Ho-induced double-strand break at the MAT locus is lethal in Candida glabrata
Source: PLoS Genet. 2020 Oct 15;16(10):e1008627. doi: 10.1371/journal.pgen.1008627 (PMC7591073; doi:10.1371/journal.pgen.1008627)

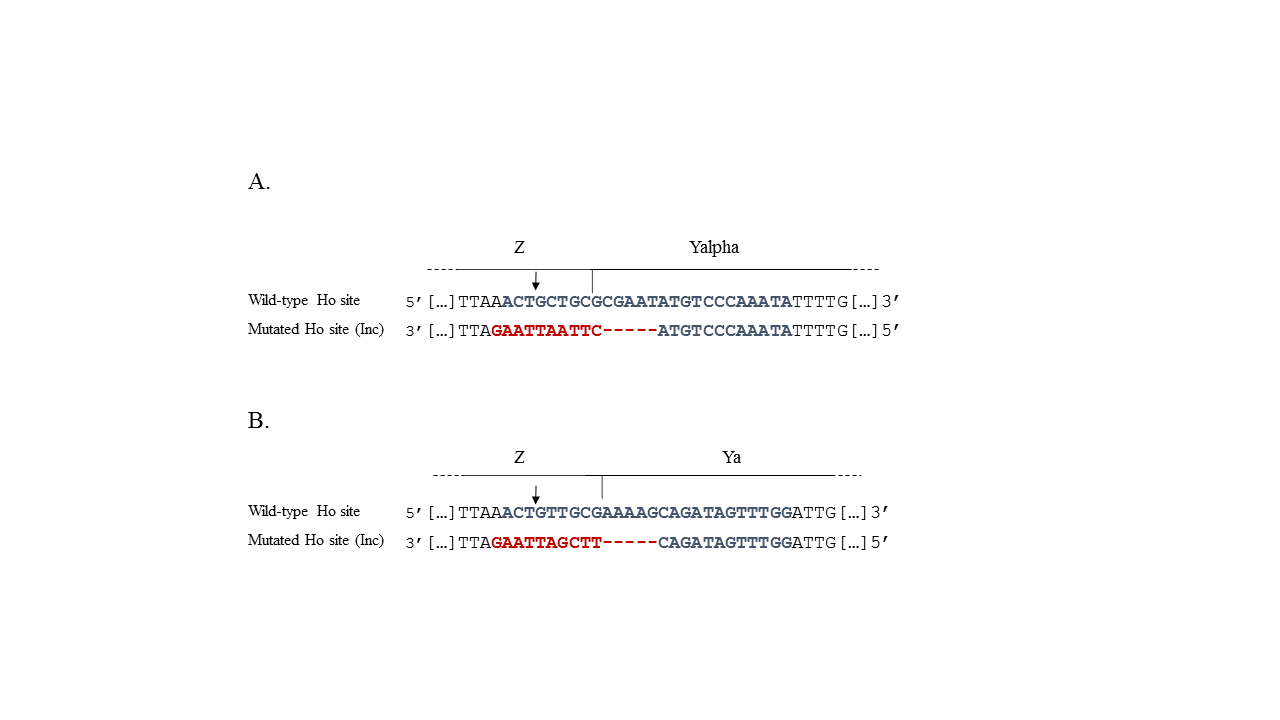

Supplement: S1 Fig — Comparison of wild-type and mutated Ho sites of loci carrying Yalpha (A) or Ya information (B). The wild-type Ho site is shown on top in blue letters, the mutated Ho site is shown below with mutated bp in red and deleted bp as dashes. Arrows indicate the Ho cleavage site. (TIF) [file pgen.1008627.s001.tif]

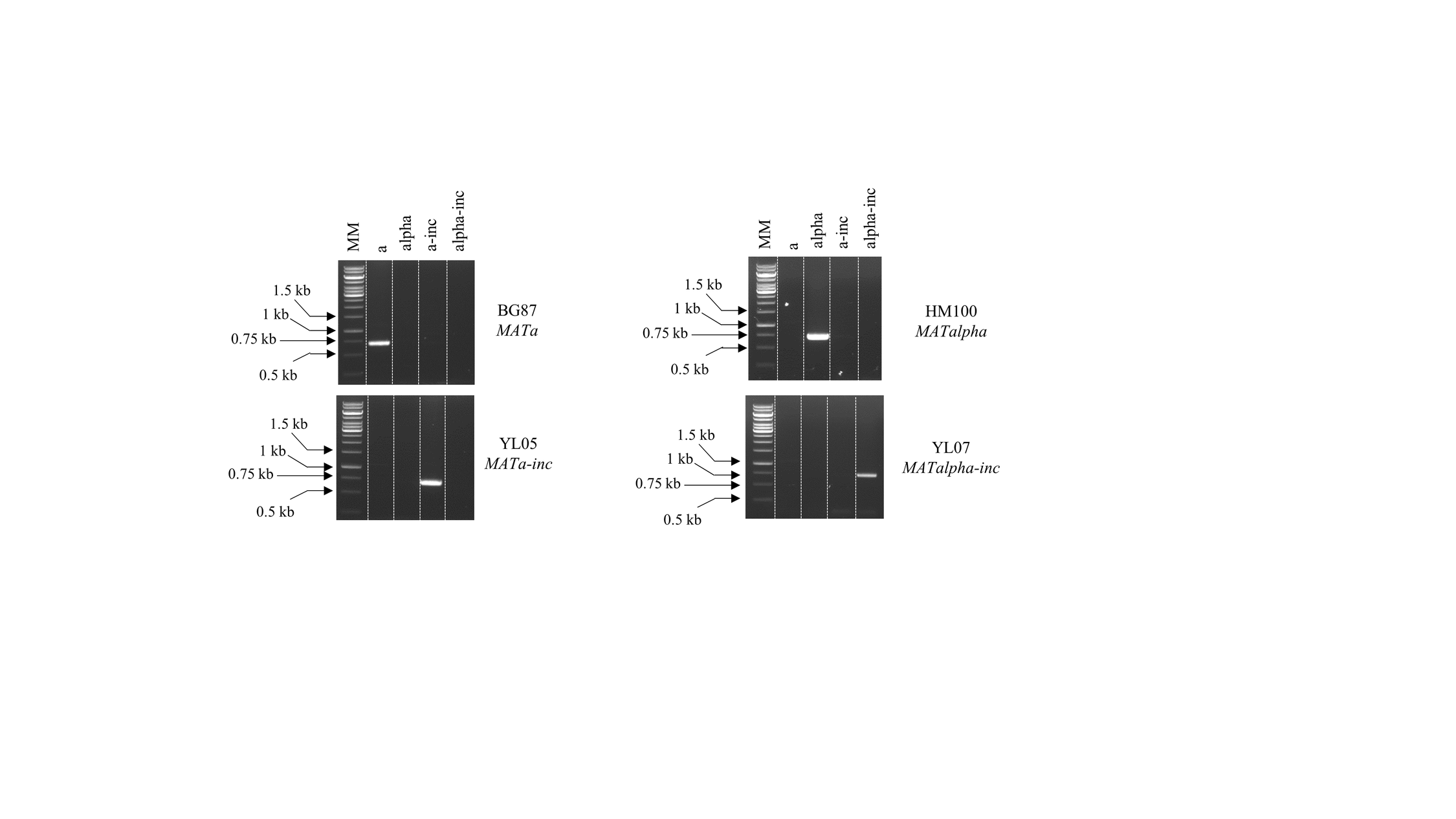

Supplement: S2 Fig — All strains are analyzed with primer pairs that are specific to MATa, MATalpha, MATa-inc and MATalpha-inc, respectively GS01/123, GS01/121, GS01/122 and GS01/120. Top left panel: amplification obtained on BG87 (MATa); bottom left panel: amplification obtained on YL05 (MATa-inc); top right panel: amplification obtained on HM100 (MATalpha); bottom right panel: amplification obtained on YL07 (MATalpha-inc). MM: Molecular Marker, GeneRuler 1 kb (Thermo Fisher Scientific Inc). (TIF) [file pgen.1008627.s002.tif]

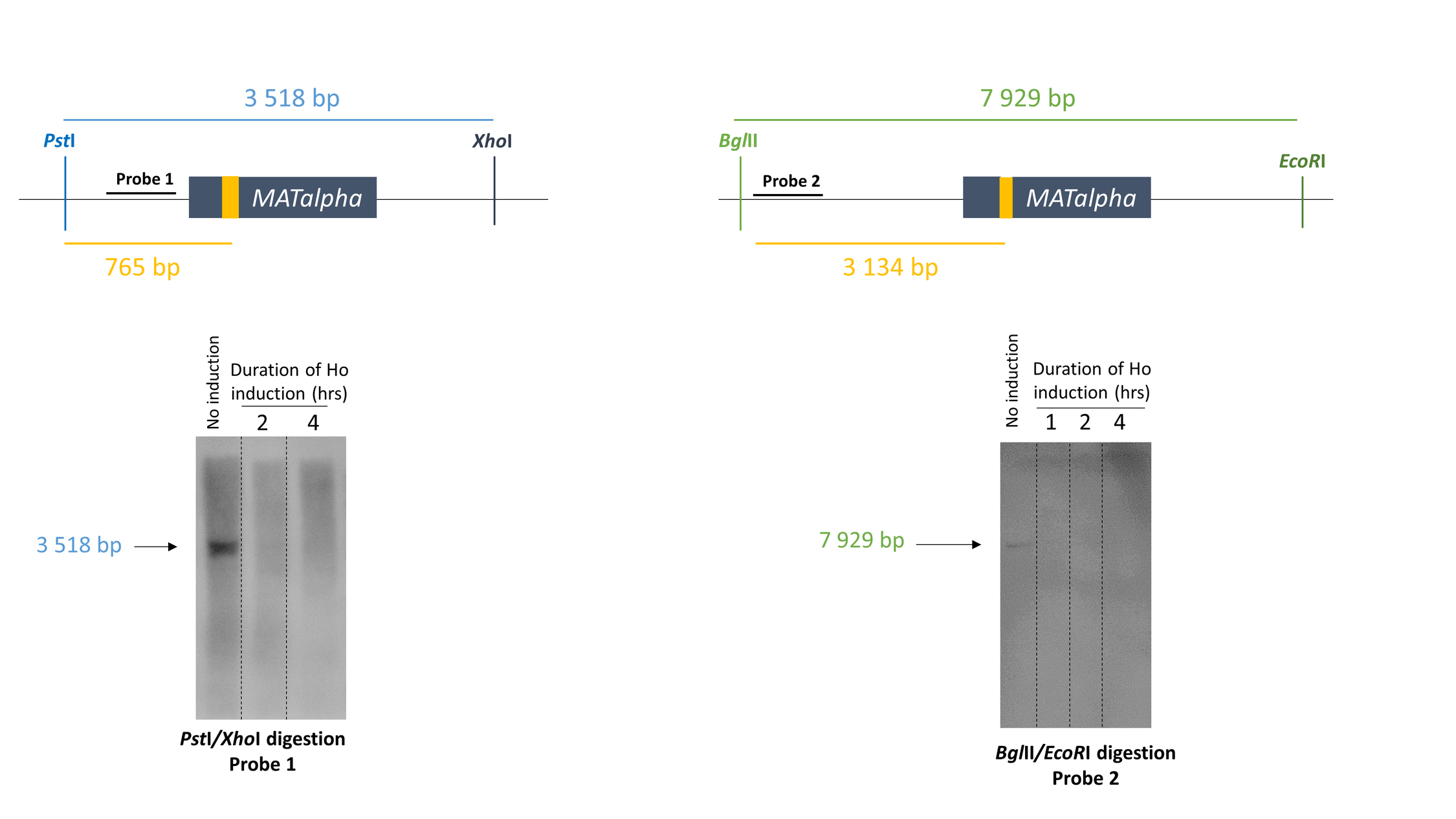

Supplement: S3 Fig — Left panel: chemiluminescence image of blot of PstI/XhoI digestion hybridized with a probe corresponding to 484 bp located 262 bp away from the first nucleotide of the Ho site. Diagram of probe in top middle panel. The regions are represented with restriction sites and size of expected fragment. Right panel: chemiluminescence image of blot of BglII/EcoRI digestion hybridized with a probe corresponding to 1,013 bp located 2,119 bp away from the first nucleotide of the Ho site. Diagram of probe is in lower middle panel. (TIF) [file pgen.1008627.s003.tif]

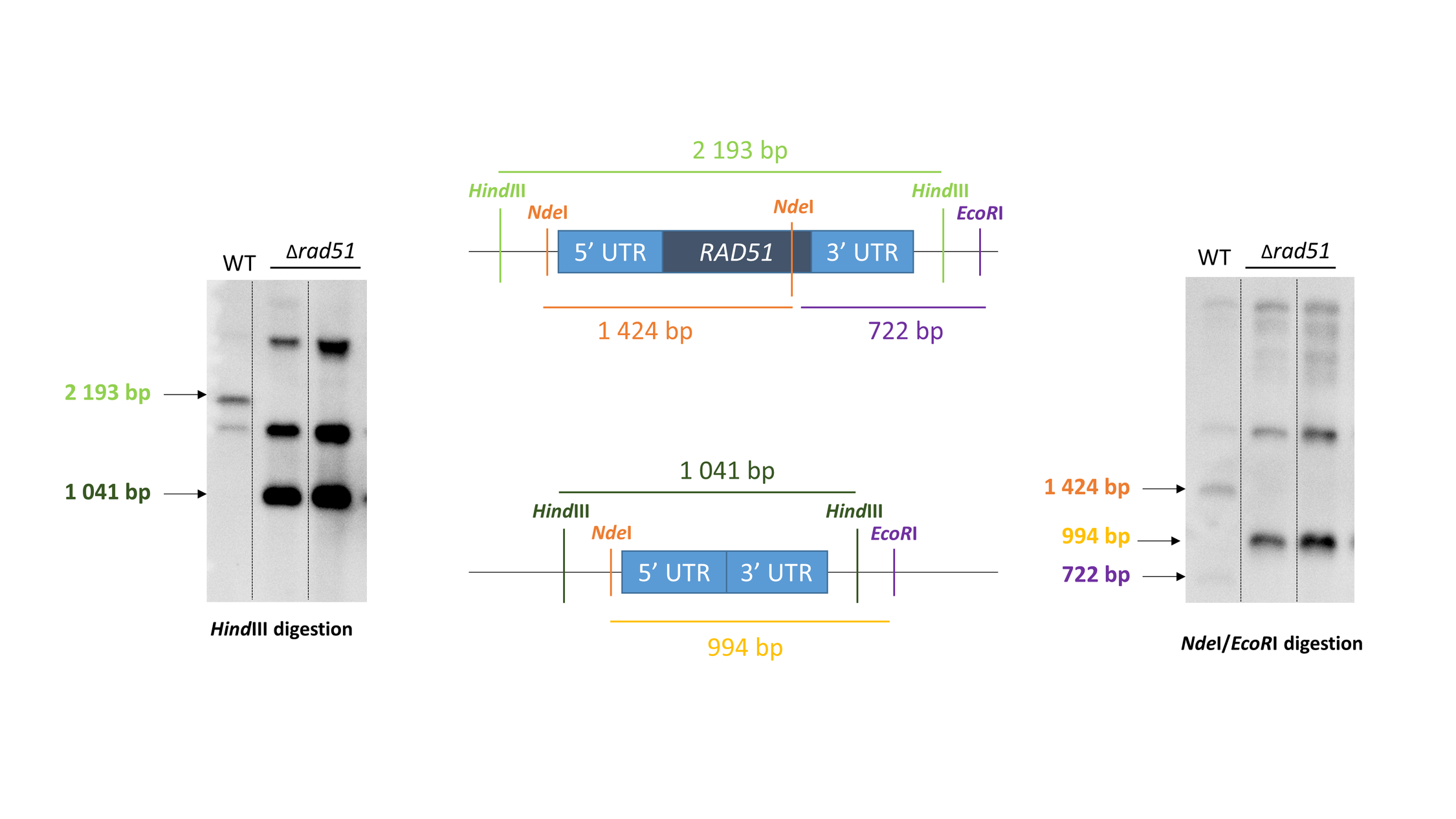

Supplement: S4 Fig — Left panel: chemiluminescence image of blot of HindIII digestion. Right panel: chemiluminescence image of blot of NdeI/EcoRI digestion. The probe used is 1 kb long and is composed to the 500 bp upstream of the RAD51 ORF fused to the 500 bp downstream of the RAD51 ORF. (TIF) [file pgen.1008627.s004.tif]
