# Supplementary material for: A single Ho-induced double-strand break at the MAT locus is lethal in Candida glabrata
Source: PLoS Genet. 2020 Oct 15;16(10):e1008627. doi: 10.1371/journal.pgen.1008627 (PMC7591073; doi:10.1371/journal.pgen.1008627)
Supplement: S1 Table — Fw: Forward; Rv: Reverse. The lowercase letters represent sequences with no homology to template DNA and reverse complete regions are indicated in uppercase. (DOCX) [file pgen.1008627.s005.docx]

| **Primers for construction of plasmids for pop-in/pop-out** | | |
| --- | --- | --- |
| Name | Sequence (5’🡪3’) | Localization |
| Sc-URA3-F | AAGAGCTTTTCAATTCATCATT | In the promoter of *URA3*, Fw |
| Sc-URA3-R | TTAGTTTTGCTGGCCGCATC | In *URA3* CDS, Rv |
| 68 | cgggatccACTCTTTCTTTTGACGCTGAG | Z1, Fw |
| 70 | cggaattcTAATTTTGCGAGTCCTCGG | Z1, Rv |
| 73 | cgaagcttCAGATAGTTTGGATTGCAAAG | Ya, Rv |
| 72 | cggtcgacAGTGCTTCTCAATACCTGC | Ya, Fw |
| 74 | cggaattcATGTCCCAAATATTTTGATCC | Yalpha, Fw |
| 69 | cggtcgacACAAAGAGCTCCTTTGGTC | Yalpha, Rv |
| **Primers for determination of mating-type** | | |
| GS01 | TACCAAGAAGCAAGAGCCCA | Upstream of *MAT*, Fw |
| GS02 | TCTTGCGTAGTCGAGACCTC | Dowstream of *MAT*, Rv |
| GS06 | GACAGGAACATCTAAGCGAT | Upstream of *HMR*, Fw |
| GS07 | GTGGATGATTACTGGGTGGA | Dowstream of *HMR*, Rv |
| GS08 | GCTGTAGTGGCGAAAATAAG | Upstream of *HML*, Fw |
| GS09 | GATCACTTCGTAGTAGAAAAC | Dowstream of *HML*, Rv |
| LM8F | GTCTTACAATTACAGCAAGG | Upstream of *EMG1* gene, Fw |
| 120-R | TATTTGGGACATGAATTAATTC | alpha-inc Ho site, Rv |
| 121-R | TGGGACATATTCGCGCAG | alpha wt Ho site, Rv |
| 122-R | CAAACTATCTGAAGCTAATTC | a-inc Ho site, Rv |
| 123-R | TATCTGCTTTTCGCAACAG | a wt Ho site, Rv |
| **Primers for construction of *Δhmr* strains** | | |
| Up-HMR-F | GTGCCGCACTTTCAAGAA | Upstream of *HMR*, Fw |
| Up-HMR-R | tatctagtttcattcttttgctcttcactcaacgtactccCCATATCTTACCTCTAA | Upstream of *HMR*, Rv |
| Down-HMR-F | GGAGTACGTTGAGTGAAG | Downstream of *HMR*, Fw |
| Down-HMR-R | CTCTAGGATTGTATCTTGAG | Downstream of *HMR*, Fw |
| Check-HMR-F | CTACAAATGAGTTCGTGAC | Upstream of the deletion of *HMR*, Fw |
| Check-HMR-R | CACATAATGGAGGATCTAC | Downstream of the deletion of *HMR*, Fw |
| **Primers for gRNA targetting *MAT*** | | |
| RNA-guide-Ya2-F | GTTGCGAAAAGCAGATAGTTgtttt | Ya Ho site, Fw |
| RNA-guide-Ya2-R | AACTATCTGCTTTTCGCAACgatca | Ya Ho site, Rv |
| **Primers for construction of plasmids pMATa-inc and pMATalpha-inc** | | |
| Up-Rec-MAT-F | catattatagaggaagaaatacgcacgaacacgatatagaggtaaaggcgcgccgaattcCTAAATAAGTCTGGAAGGTG | Upstream of *MAT*, Fw |
| Down-Rec-MAT-R | acggccagtgagcgcgcgtaatacgactcactatagggcgaattggcggccgcgaattcCTTGCGTAGTCGAGACCTC | Downstream of *MAT*, Rv |
| **Primers for deletion of *RAD51* and Southern blot analysis** | | |
| Up-RAD51-F | AGAAAGAAGAACATTGCACC | Upstream of *RAD51*, Fw |
| Up-RAD51-R | agaacaattctccttaaaaaggtaaatatctattttacatGTTATTATTCGTTAAGGTAG | Upstream of *RAD51*, Rv |
| Down-RAD51-F | ATGTAAAATAGATATTTACC | Downstream of *RAD51*, Fw |
| Down-RAD51-R | CTGACCAATGCTCGATGAGC | Downstream of *RAD51*, Rv |
| Control-RAD51-F | CTCTGTTATACCAAGGTCTC | Upstream of the deletion of *RAD51*, Fw |
| Control-RAD51-R | GTTGAGTCCTTCTGGTGAG | Downstream of the deletion of *RAD51*, Rv |
| CDS-RAD51-F | GCGAACAGTTATTTCACC | In *RAD51* CDS, Fw |
| CDS-RAD51-R | GTCGTTGATGAATGTGCC | In *RAD51* CDS, Rv |
| **Primers for Southern-blot analysis of the *Sc*Ho-DSB at *MAT*** | | |
| Southern-MAT-F | AGATAGAAGGATAAAATAAGC | Upstream of *MAT*, Fw |
| Southern-MAT-R | GTCGGCAGGCAGGTATTTC | Downstream of *MAT*, Rv |
| Southern-MAT-2F | CCAAAAGGCTAAGAGAAGAC | Upstream of *MAT*, Fw |
| Southern-MAT-2R | AACTATGCAGAACAAGTTAGC | Downstream of *MAT*, Rv |
