# Supplementary material for: A single Ho-induced double-strand break at the MAT locus is lethal in Candida glabrata
Source: PLoS Genet. 2020 Oct 15;16(10):e1008627. doi: 10.1371/journal.pgen.1008627 (PMC7591073; doi:10.1371/journal.pgen.1008627)
Supplement: S2 Table — (DOCX) [file pgen.1008627.s006.docx]

| **Name** | **Description** | **Reference** |
| --- | --- | --- |
| **Plasmid used for pop-in/pop-out** | | |
| YEp352 | Plasmid expression for *S. cerevisiae, Ura3+* | [1] |
| pURA | *S. cerevisiae* *URA3* cloned into pBlueScript Amp^R^ | This work. |
| pZU | Z sequence cloned upstream of *URA3* into pURA Amp^R^ | This work. |
| pZUA | Ya sequence cloned downstream of *URA3* into pZU Amp^R^ | This work. |
| pZUalpha | Yalpha sequence cloned downstream of *URA3* into pZU Amp^R^ | This work. |
| pZA-inc | pZUA digested by *EcoR*I/*Hind*III and religated after a Klenow treatment | This work. |
| pZAlpha-inc | Z sequence and truncated version of Yalpha sequence cloned into pBlueScript, Amp^R^ | This work. |
| **Plasmids for *S. cerevisiae’s HO* gene expression in *C. glabrata*** | | |
| pYR32 | Plasmid expression for *C. glabrata* Ura3+ | [2] |
| p7.1 | *S. cerevisiae*’s *HO* gene cloned under inducible *C. glabrata* *MET3* promoter into pYR32 Ura3+ | [3] |
| pMATa-inc | *MATa*-*inc* locus cloned into p7.1 Ura3+ | This work. |
| pMATalpha-inc | *MATalpha*-*inc* locus cloned into p7.1 Ura3+ | This work. |
| **Plasmids for *CAS9/gRNA* expression in *C. glabrata*** | | |
| pJH2972 | CRISPR-Cas9 vector Ura3+ | https://protocolexchange.researchsquare.com/article/nprot-5791/v1 |
| pJH2972-RAD51 | Sequence coding a gRNA targeting *C. glabrata RAD51* cloned into pJH2972 Ura3+ | This work. |
| pCGLM1 | Inductible CRISPR/Cas9 vector Ura3+ | [4] |
| pCGLM1-Ya2 | Sequence coding a gRNA targeting Ya cloned into pCGLM1 Ura3+ | This work. |

1. Hill JE, Myers AM, Koerner TJ, Tzagoloff A. Yeast/E. coli shuttle vectors with multiple unique restriction sites. Yeast. 1986;2: 163–167. doi:10.1002/yea.320020304

2. Zordan RE, Ren Y, Pan S-J, Rotondo G, De Las Peñas A, Iluore J, et al. Expression plasmids for use in Candida glabrata. G3 (Bethesda). 2013;3: 1675–1686. doi:10.1534/g3.113.006908

3. Boisnard S, Zhou Li Y, Arnaise S, Sequeira G, Raffoux X, Enache-Angoulvant A, et al. Efficient Mating-Type Switching in Candida glabrata Induces Cell Death. PLoS ONE. 2015;10: e0140990. doi:10.1371/journal.pone.0140990

4. Maroc L, Fairhead C. A new inducible CRISPR-Cas9 system useful for genome editing and study of double-strand break repair in Candida glabrata. Yeast. 2019. doi:10.1002/yea.3440
