# Supplementary material for: A single Ho-induced double-strand break at the MAT locus is lethal in Candida glabrata
Source: PLoS Genet. 2020 Oct 15;16(10):e1008627. doi: 10.1371/journal.pgen.1008627 (PMC7591073; doi:10.1371/journal.pgen.1008627)
Supplement: S4 Table — (DOCX) [file pgen.1008627.s008.docx]

A.

| **Strains constructed by pop-in/pop-out** | | | | |
| --- | --- | --- | --- | --- |
| **Name and genotype** | **Parental strain** | **Target locus** | **Pop-in**  **PCR-Fragments** | **Pop-out**  **PCR-Fragments** |
| YL01  *HMLalpha MATalpha HMRa-inc* | HM100 | *HMR* | ZUA | ZA-inc |
| YL02  *HMLalpha-inc MATalpha HMRa* | HM100 | *HML* | ZUAlpha | Zalpha-inc |
| YL03-MATalpha  *HMLalpha-inc MATalpha HMRa-inc* | YL02 | *HMR* | ZUA | ZA-inc |
| YL03-MATa  *HMLalpha-inc MATa HMRa-inc* | YL03-MATalpha | *MAT* | ZUAlpha | *MATa* (amplified with primers  GS01/GS02 on BG87) |
| YL04  *HMLalpha MATalpha-inc HMRa* | YL07 | *HML* | ZUAlpha | Zalpha |
| YL05  *HMLalpha MATa-inc HMRa-inc* | YL09 | *HML* | ZUA | *HMLalpha*  (amplified with primers GS08/GS09 on HM100) |
| SL01  *HMLalpha* *MATa* *Δhmr* | BG87 | *HMR* | ZUA | *Δhmr* |
| SL-CG1  *HMLalpha* *Δmat Δhmr* | CGM390 | *HMR* | ZUA | *Δhmr* |
| SL-CG8  *HMLalpha-inc* *MATa* *Δhmr* | SL01 | *HML* | ZUAlpha | *HMLalpha*-inc (amplified with primers GS08/GS09 on YL03-MATa) |
| SL-CG9  Δ*hml* *MATa* *HMRalpha-inc* | CGM460 | *HMR* | ZUA | *HMRa*lpha-inc (amplified with primers GS06/GS07 on YL10) |

B.

| **Strains constructed by Ho mating-type switching** | | |
| --- | --- | --- |
| Name | Parental strain | Template used for switch |
| YL07  *HMLalpha-inc MATalpha-inc HMRa* | YL02 | Genomic *HMLalpha-inc* |
| YL09  *HMLa-inc MATa-inc HMRa-inc* | YL01 | Genomic *HMRa-inc* |
| YL10  *HMLalpha-inc MATalpha-inc HMRalpha-inc* | YL07 | Genomic *HMLalpha-inc* or *MATalpha*-*inc* |
| SL09  *HMLa-inc MATa-inc HMRa-inc* | BG87 | Plasmidic *MATa-inc* |
| SL-CG10  *HMLa-inc MATa-inc Δhmr* | SL01 | Plasmidic *MATa-inc* |
| SL-CG12  *HMLalpha-inc* *Δmat HMRalpha-inc* | CGM390 | Plasmidic *MATalpha-inc* |
| SL-CG14  *Δhml MATalpha-inc HMRalpha-inc* | CGM460 | Plasmidic *MATalpha-inc* |
| SL0A  *HMLalpha MATalpha-inc HMRa-inc* | YL01 | Plasmidic *MATalpha-inc* |
| SL0B  *HMLa-inc MATalpha-inc HMRa* | YL04 | Plasmidic *MATa-inc* |
