# Supplementary material for: A single Ho-induced double-strand break at the MAT locus is lethal in Candida glabrata
Source: PLoS Genet. 2020 Oct 15;16(10):e1008627. doi: 10.1371/journal.pgen.1008627 (PMC7591073; doi:10.1371/journal.pgen.1008627)
Supplement: Attachment — Submitted filename: Reply to reviewers PLoS Genet v2cf.docx [file pgen.1008627.s009.docx]

Reviewer's Responses to Questions

**Comments to the Authors:
Please note here if the review is uploaded as an attachment.**

Reviewer #1: This is a careful analysis of the fate of Candida glabrata cells when the Ho endonuclease is expressed in Candida. An earlier publication showed that expression of Ho generated DSBs that were substrates for recombination and mating type switching but that resulted in very drastic lethality. The result shown here by analyzing switching and lethality in mutant strains where Ho sites have been removed from MAT, HML or HMR is that cleavage at MAT alone is sufficient to generate lethality independent of the mating type information at MAT. The data are consistent with the DSB being poorly repaired when generated by Ho, since a DSB generated by CRISPR/CAS show low lethality.

The authors nicely show that lethality depends on having a Ho-sensitive MAT allele. Interestingly, the survival does not increase when both HMR and HML are Inc. This suggests that cleavage at these loci is not an important contributor to lethality but also that repair from these loci and then persistent re-cutting is probably not the mechanism of lethality since repairing with an INC substrate would be expected to increase survivability. Rather the data suggest that the cut at MAT is not easily repairable. Cutting at MAT by CRISPR/CAS results in efficient switching but no lethality. In colonies that result, 80-100% of cells have switched and are viable. This suggests that the DSB generated by Ho is specifically lethal.

These data are excellent and the conclusions are compelling and the genetic analysis with the engineered strains is elegant and informative, and the results are surprising. Some additional controls are needed, or if they exist should be shown, to see whether cleavage by Ho is occurring at HMR and HML. If these are actively being cut and gene converted by the other HM locus or by MAT, then the question is why there is no lethality. Are the survivors in YL04 YL05 and YL07 able to repair a DSB, or is the information between HML and HMR non-essential and simply lost, or are these loci not being cleaved at appreciable rates? The manuscript does not present quantitative data to show whether there is cleavage at HML and HMR and whether DSBs there are repaired by homologous recombination. This would help distinguish models for why MAT DSBs are so toxic.

We thank the reviewer for her/his suggestions and we have included the experiments asked. We first thought that it would have been easier to focus this paper on the Ho-DSB at MAT and its impact on cell survival and to write a second paper on the study of the Ho-DSB at HML and HMR. We now agree with the reviewer that such results are missing in our manuscript and we hope that their inclusion will greatly improve our manuscript.

The inclusion of the results modifies extensively the structure of the paper. Specifically, to address the concerns of reviewer 1, Table 2 has been modified to integrate molecular analysis of strains HM100, YL05 and YL07; Table 1 has been modified to add strains SL-CG1 and CGM498 and CGM390, which demonstrate that the Ho-cut at HML and HMR is efficient. This was not presented in the first submitted manuscript.

The paper is a little anticlimactic since there is no final conclusion as to why the Ho generated DSB can't be repaired. it would be easily improved by direct assays demonstrating persistence of the DSB. If it is happening in 99% of cells, can the DSB be visualized by Southern, or quantitated by a PCR assay with a primer ligated to the break? If so, comparing DSB persistence at MAT HML and HMR would add significantly to the paper. Can a tagged version of Ho be used for an IP to show presence at the MAT locus and/or the HML and HMR loci?

We regret that we were unable to discover why the Ho-DSB is lethal at MAT. We performed a Southern-blot analysis of the DSB at MAT in the wild-type HM100 strain (now S3 Fig). We used a probe adjacent to MAT. Without Ho induction, only the band corresponding to the intact MAT locus is detected. Upon Ho induction, we never observe a band corresponding to the MAT locus cut by Ho, whatever the duration of induction. We performed a second Southern-blot analysis with a probe located further than the first one from the Ho cut. We obtained exactly the same result as with the first Southern-blot. These two Southern-blots have been added as a S3 Fig and are discussed in the manuscript.

We have indeed planned new experiments to investigate binding (and perhaps persistence) of the ScHo protein at MAT but also at HML and HMR by a ChIP-PCR, using a GFP-tagged Ho. However, we are aware that performing a ChIP experiment on an endonuclease is very risky and we plan on including results in another paper, if successful.

We have rewritten the discussion of the paper in order to present our hypotheses and ideas. We hope that it is now clearer.

Reviewer #2: This is an intriguing and well written manuscript that aims to understand the mechanism by which the Saccharomyces cerevisiae (Sc) HO endonuclease causes severe lethality when expressed in Candida glabrata (Cg) cells. It has implications for our understanding of how HO works, and the manuscript provides some interesting indications that HO has a function that goes beyond being a simple endonuclease.

In previous work (Boisnard et al 2015, ref. 22) the authors’ group discovered that expressing Sc HO in C. glabrata causes a high rate of cell death (99.9% of cells die). In that study, they proposed that the lethality was caused by simultaneous cutting of the Cg HMR and HML loci as well as the MAT locus, leading to absence of intact templates for DNA repair. However, in the current manuscript Maroc et al test this hypothesis and reject it. In a careful series of experiments in which they mutated the HO cleavage sites in Cg MAT, HML and/or HMR, they show that HO-cleavage of the MAT locus alone is sufficient to cause lethality, even though most of the few cells that survive HO expression have switched mating type. Maroc et al also show that, in contrast, cleavage of the Cg MAT locus by CRISPR-Cas9 does not cause lethality and leads to mating-type switching. So the lethality does not stem from the existence of a DSB per se, but from the existence of a DSB in association with HO, which is intriguing. Although the manuscript does not succeed in its goal of identifying the molecular cause of the incompatibility between Sc HO and Cg MAT, it goes a long way towards narrowing down the range of possible causes.

Major comment

L368-378 (end of Discussion section): The manuscript has made a substantial step towards understanding the toxic role of S. cerevisiae HO in C. glabrata, but we are left with the possibility that HO might be toxic because it attacks some unknown off-target (non MAT) site in the C. glabrata genome (L372-373). A fairly simple way to address this question experimentally would be to see if the lethality can be moved into S. cerevisiae, by moving the Cg MAT cleavage site into S. cerevisiae. Construct a haploid S. cerevisiae strain that is uncleavable at the Sc MAT locus (MAT-inc or similar), but which also carries the Cg cleavage site (~100 bp spanning the Cg Y/Z junction) somewhere in its genome, and then introduce HO from p7.1 to see if it causes lethality.

We thank the reviewer for her/his comments. The first concern of the reviewer is that “that HO might be toxic because it attacks some unknown off-target (non MAT) site in the C. glabrata genome (L372-373).”. We have included further experiments in an additional paragraph at the end of the Results section (“Exploring the residual lethality in the absence of MTL-cuts” lines 417-440). We believe that it contradicts the hypothesis of a lethality due to off-targets.

The reviewer proposed to move our experiments to S. cerevisiae and suggests that it is the combination of ScHO and the Ho site of C. glabrata that it lethal. Although the experiment proposed would be very interesting, we have shown that the identical Ho site at HML and HMR does induce massive lethality upon Ho-cutting. Indeed, we believe it is the combination of C. glabrata with the Ho cleavage site specifically at MAT that is lethal.

Minor comments

L24, L28, The taxonomic group can be called either Ascomycota (phylum) or Saccharomycotina (subphylum), but there is no ‘Ascomycotina’.

We thank the reviewer for noting this, the term has been changed for Saccharomycotina.

L40, add “…, whereas one DSB at HML or HMR is not.”

It has been added.

L44, change to “…, but that repair is prevented by S. cerevisiae’s Ho”.

It has been changed.

L86, change to “… mat1, called mat2 and mat3”.

It has been changed.

L88, also cite Rajaei et al, PMID 25313032.

It has been added.


L100, change “implies” to “involves”

It has been changed.

L114, L221, L305: When referring to the RE, the manuscript cites reference 15 (a review article by Herskowitz 1988) several times. Surely there are more relevant references, , such as PMID 8861911, 9335581.

They have been changed for reference Wu et al., 1996 PMID 8861911.

L132-133: change to “a series of inconvertible (inc) C. glabrata strains … DNA breaks during induction of S. cerevisiae Ho … “

It has been changed.

L152: I was surprised by the reference to data “not shown” here, because the title of this section is “HMR is cut by Ho …”. The authors do show data in support of HMR cleavage later in the manuscript (SL-CG9 results in Table 2) so it would be preferable to rearrange the order of the sections in Results / or merge some of them / or rephase L152 to say that evidence for cleavage of HMR will be presented in a subsequent section of the paper.

We thank the reviewer for her/his comments. Data showing HMR cleavage have been added to manuscript in Table 2 and “not shown” has been replaced by “(Table2)”.

L232: This paragraph should not begin with “Similarly...” but “In contrast,… ” because the frequency of switching in the correct direction changes dramatically from 97% (MATa to MATalpha) to <14% (MATalpha to MATa).

We had used the word “similar” because although the frequency of switching is indeed dramatically different, the use of HML template is similar for both MATalpha and MATa Ho-cut. For the sake of clarity, we have deleted this part of the sentence.

L300: change to “cut by the S. cerevisiae Ho endonuclease”

We have now inserted ScHo in every instance.

L311-315: I agree with the authors that the mating-type switching system in C. glabrata is less active/efficient than in S. cerevisiae, and that the word “degenerated” is probably an accurate word to use. However Cg HO is not a pseudogene, which is what we would expect if it truly has no function. One could speculate that HO might have a second function in addition to being the mating-type switching endonuclease, and this second function causes the HO gene to be maintained in C. glabrata. In the population genomics data of Carrete et al (2018), did all the studied C. glabrata stains have an intact HO ORF, and was the nonsynonymous/synonymous substitution ratio for this gene <1, indicating purifying selection ?

We agree with the reviewer’s remark and believe that the Ho protein might have a second function that has not been discovered yet and for which HO has been conserved in C. glabrata. Carreté et al (2018) show that the HO gene is under purifying selection as it has a nonsynonymous/synonymous substitution ratio of 0,126126126 that is even lower that the one in S. cerevisiae (0,454545455). We included this remark at the end of the Discussion section.

L350: Change to “suggest that the S. cerevisiae Ho protein prevents DSB repair specifically at the MAT locus of C. glabrata”

It has been changed.

L357-359. I didn’t understand the sentences “We know that mating-type borne by any of the MAT does not influence lethality as both HM100 and BG87 die at 99.99 % (Fig 1). Thus, only the location of the MAT locus could explain the specificity of lethality induced by Ho.” Do you mean “We know that lethality occurs in both MATa and MATalpha cells, because both HM100 and BG87 die at 99.99%”?

We have modified the sentence to “We know that mating-type does not influence lethality in any of our experiments”.

L361: ref 36 is the wrong reference.

Reference has been corrected to Muller et al., 2007.

L368: “Finally, we would like to discuss the toxic role of S. cerevisiae’s Ho, in C. glabrata, outside its role in lethal DSB at MAT.” Is this an hypothesis (you are proposing that it has an unidentified toxic role apart from DSB formation), or is it a statement of fact? If it is a statement of fact, what is the evidence that such a toxic role outside DSB formation exists?

We thank the reviewer for her/his comments. We have extensively modified the manuscript to include many more strains with corresponding experiments. We have also added a final paragraph to the Results section, dedicated to these results; and they are discussed in the Discussion section.

The plasmid name p7.1 appears in the titles of several Figures and Tables, but it is not explained in the text until line 402. It should be introduced on approx. line 180.

We thank the reviewer for having noticed this. We have corrected this.

Figure 1: Shouldn’t HML be colored red (alpha) in HM100 and HM100 Δrad51 ?

We thank the reviewer for having noticed this. Color has been changed for red.

Figure 2: I found the X-axis title “Time of nuclease induction” confusing because it seems to indicate that the nuclease was induced at time T, where T = 0, 2, 4 hrs etc. But I think that you actually mean that the nuclease was induced at T=0 and then repressed at T = 2, 4, hrs etc. Change the title to “Duration of nuclease induction” ?

We thank the reviewer for this remark, and we agree with her/him. We changed the title of the X-axis to “Duration of nuclease induction”.

Figure 3B: The HO cut is shown as a blunt DSB, but it should be a 4-bp 3’ overhang. Also, the Y/Z junction is drawn in the wrong place: it should be between the TTTT and the CGCAACA on the bottom strand (if we permit a 1-bp mismatch in the Z region, CGCAACA in MATa and CGCAGCA in MATalpha).

We thank the reviewer for having noticed this. It has been corrected in all related figures (Fig 3 now Fig 5 and now S1 Fig).

Tables 2 and 3: For readers like me who are slow at mental arithmetic, it would be easier to relate the colony numbers in the Tables to the percentages in the text, if you showed percentages as well as numbers in the Tables. For example, for the SL-CG9 Cas9 experiment, Line 264 says that 87% of colonies presented MATalpha-inc and cites Table 3, but to find this information in Table 3 I have to calculate that 32/45 + 7/45 = 87%.

We thank the reviewer; we have added a column corresponding to the percentage of switch for each strain in Table 2 and Table 3.

Reviewer #3: Mating type switch in Saccharomyces and related yeasts is a well-studied process, but many unanswered questions remain. The authors have addressed the puzzling retention of the mating-type switching apparatus in Candida glabrata, even though it is apparently asexual. Intriguingly, they showed that that they can induce switching in C. glabrata using CRISPR-Cas9, demonstrating that the all the components (apart from the endonuclease) are fully functional. However, when the S. cerevisiae HO endonuclease is overexpressed, cutting at MAT results in cell death. HML and HMR are also cut. These experiments are carefully performed, but the underlying biological mechanism effect is not clear.

1. I think that the CRISPR-Cas9 mediated induction of switching is one of the most interesting parts of this paper. It has been shown in S. cerevisiae, but this is the first time that it is clear that switching is fully functional in C. glabrata (apart from the endonuclease) and that cells can switch without inducing cell death. I suggest re-framing the paper, and starting from here.

We thank the reviewer for her/his suggestion. The main goal of this paper was to understand why the induction of mating-type switching by S. cerevisiae’s Ho leads to a high lethality in C. glabrata. Even though we also believe that the CRISPR-Cas9 mediated induction of switching is a major result, we wish to put the focus on Ho. To this effect, we have added many new experiments with Ho.

2. RAD51 should be inactivated in the Cas9-activated strain, this would show more clearly if switching relies on homologous recombination.

We thank the reviewer for her/his suggestion. Unfortunately, due to the present conditions, we were unable to do this experiment before re-submitting the paper. Nonetheless, we believe the new data we have included shows that switching relies on homologous recombination: the molecular results of each MTL locus in strain HM100, upon Ho induction, clearly shows that almost a 100 % of surviving colonies showed a mating-type switching at each MTL locus whereas in the HM100 Δrad51 not even one surviving colony shows switching at one MTL locus. Furthermore, we have added in our manuscript results obtained at HML and HMR. In strain CGM498 (Δhml Δmat HMRa) and SL-CG1 (HMLalpha Δmat Δhmr) survival, upon Ho induction, is comparable to the one obtained in the wild-type strain, showing that without any repair template, an Ho-DSB at HML and HMR becomes lethal.

3. The authors show that Cas9 induces switching but not cell death, whereas expression of ScHO induces switching and cell death. They suggest that Ho may remain at the cut ends. What happens if Cas9 and Ho are added/induced at the same time? This might help to work out the mechanism.

It is unclear to us whether the reviewer suggests inducing cutting at the same site as Ho. Furthermore, we cannot, for the moment, induce Ho and Cas9 at the same time for lack of auxotrophic markers.

4. I think that the observation that expressing ScHO in K. delphensis does not induce cell death is important, and should be shown in the manuscript.

This result will be soon published in an article named “The complete sexual cycle of Nakaseomyces delphensis and the role of Ho in ploidy” which is under review. We have removed this observation from our manuscript, since it is not allowed to cite data not shown.

5. There seems to be an error in Fig. 1, the colors for the top two strains are not correct, surely they are HMLalpha?

The top two strains are indeed HMLalpha and we first colored HML with the wrong color. It has been corrected (we changed the color from blue to red) on now Fig 2.

Minor points

1. The start of the results section is hard to follow. It is assumed that the reader is very familiar with the earlier experiments. More details, for example how ScHO is expressed and how the target sites are changed, would be helpful. Switching at HMR should be shown, PLoS does not allow “data not shown”

We thank the reviewer for her/his comments. More details about the method used for induction and about earlier experiments have been added at the beginning of the results section. Data showing HMR cleavage has been added to the manuscript in Table 2 and “not shown” has been replaced by “(Table 2)”. We have also added a whole paragraph in the Results section about switching at HML and HMR. We substantially changed the structure of the paper and we hope that it is clearer now.

2. There are some odd phrases. Line 138 might be better phrased as “ We find that even when HML and HMR are protected, introducing a DSB at MAT is sufficient…”

This has been corrected.

3. Line 124 has an extra “to”.

It has been deleted.

4. Line 128: unlike, rather than contrary

It has been corrected.

5. Line 85: Clarify what an “imprint” at mat1 means.

It has been clarified.

Reviewer #4: The manuscript ‘A single HO-induced double-strand break at the MAT locus is lethal in Candida glabrata’ seeks to follows up on an interesting result from a previous study that found lethality associated with heterologous S. cerevisiae HO expression in C. glabrata. The authors find that the rare cells that survive ScHO expression can indeed undergo mating type switching, although there is a preference for HML over HMR, regardless of the MAT genotype. Furthermore, mating type switching is dependent on Rad51 and homologous recombination. The authors construct a series of strains in which they disrupt the HO-cleavage sites at the MAT or cryptic mating type loci, HML and HMR and then measure cell lethality when cutting is blocked. Intriguingly, it is cleavage at the MAT locus, but not the cryptic mating type loci that results in a lethal phenotype. Furthermore, MAT-cleavage lethality is specific to HO cutting – the authors induce CAS9 cleavage at the MAT locus and find that lethality is abolished, but the frequency of mating-type switching is reduced compared to HO cleavage. While this is an interesting insight into the degradation of pseudo-homothallism in C. glabrata, the current presentation and organization of this manuscript makes it difficult to follow and does not provide enough context for non-expert readers, and the interpretation of the HO-specific lethality is tenuous, given the limitations of the experiment. Taken together, this work is not of sufficient interest to the broad range of PLoS Genetics readers in its current state. Furthermore, there are several experimental considerations that should be taken into account.

1) There are currently three main figures – but many aspects of figures 1 and 2 and not discussed until much later in the manuscript. As a reader this was very difficult to discern what was going on. I suggest rearranging the figures. The first part of figure 1 (all the strains that have intact cleave sites) should be presented with figure 2a. The second figure should be the constructed strains that cannot cut at the various loci and their resulting survival. The third figure should be regarding the Cas9-induced cleavage and include figure 2b (which is currently not discussed or referred to in the text).

We thank the reviewer for her/his comments. We have rearranged the structure of the manuscript as well as the figures, as suggested.

2) The CAS9-induced cleavage is a very cool experiment, but given that the cleavage site is different, if only by an 18-bp shift, it is hard to directly compare HO- and CAS9- induced cleavage. If the authors designed additional gDNAs that could cut at many different places in the MAT locus this would bolster their argument that lethality is specific to HO. Alternatively, if the HO cleavage site was moved to different locations within the MAT locus and still resulted in lethality, this also would better support that HO-cleavage is distinct from other DSBs and not due to exact location of the cut.

We thank the reviewer for her/his comments. We had designed three additional gDNAs to direct the Cas9-cut at the MAT locus including one that allows a Cas9-cut only 1-bp away from the HO site. Experiments using these three gRNA have been included in the manuscript in Fig 5, which has been modified consequently. Unfortunately, only the gRNA Ya2 presented in the first version of the manuscript, allows an efficient Cas9-DSB and thus a full comparison with the Ho-DSB. This gRNA is, in fact, 14-bp away from the Ho site (there was an error in the Ho cut in the first version of this manuscript).

3) The authors refer to cell viability as the survival rate, but my interpretation of the experiment is that this is a frequency of surviving cells relative to the total number in the population, and thus it would be more appropriate to refer to this as a frequency, or simply % survival.

We thank the reviewer for her/his comments. We changed Survival rate by “% survival” on our figures.

4) I’m a little confused about the ‘mixed’ mating-type analysis – does this suggest that some surviving cells are able to survive because they are delayed in HO cleavage and thus, when HO cuts it can switch using HML or HMR within that colony? Also, the sub-cloning results seemed inconsistent with the original analysis. The original analysis showed that ~20% of colonies were mixed, but then the subcloning indicated that there were no MATa-inc isolated? Clarification regarding this experiment and analysis is needed.

Indeed, mixed surviving colonies must arise from distinct repair events occurring in at least two different cells. Screening by PCR is a very sensitive method and can thus detect rare events, which probably would have been isolated if we had sub-cloned hundreds of colonies.

We feel that discussions about mixed colonies are indeed confusing for the reader, and we have decided to omit sub-clones from all Ho experiments. We have calculated the percentage of switch as suggested by reviewer number 2 without taking into account mixed colonies since we think the results are clear-cut without them. In the case of the Cas9 experiments, we have performed sub-cloning experiments because results are less clear-cut and because it was the first time that we performed this experiment. We hope this will satisfy the reviewer.

5) I’m not exactly sure what audience this paper was written for. Certainly for an expert audience in yeast mating-type switching, but aside from that, it’s unclear if the scope of the paper is to investigate the molecular mechanism by which switching is lethal in C. glabrata or to investigate the evolutionary trajectory as to why C. glabrata is seemingly asexual. I think it is likely both, but these ideas are not woven together and developed in this manuscript. Most experiments in the results are not introduced with the specific question they are designed to address, which leaves the reader guessing as to what we are trying to learn. Providing more background and context for the motivation for this study (beyond just stating that an earlier study showed heterologous expression of ScHO is lethal in C. glabrata) is needed.

We have substantially modified the manuscript, in particular, we have tried to state the specific question asked at the beginning of all Results sub-sections. We hope that it is now clearer. As for the scope of the paper, we focus on the apparent degeneration of the switching system in C. glabrata. We have tried to link this to asexuality, in the Introduction section and in the last paragraph of the Discussion section.
