# Supplementary material for: A single Ho-induced double-strand break at the MAT locus is lethal in Candida glabrata
Source: PLoS Genet. 2020 Oct 15;16(10):e1008627. doi: 10.1371/journal.pgen.1008627 (PMC7591073; doi:10.1371/journal.pgen.1008627)
Supplement: Attachment — Submitted filename: Response to Reviewers.docx [file pgen.1008627.s010.docx]

Reviewer #1: This revision appropriately addresses many reviewers' concerns. Demonstration of efficient cleavage and repair at HML and HMR adds significantly to the paper. It highlights the the key question of why HO generated breaks at MAT are lethal while cleavage at HML or HMR is non lethal as long as there is a template to repair from.

To limit the reasons for this locus specific lethality, an additional experiment would be helpful.

Replace the entire HML or HMR locus with the entire MAT locus, and determine if cleavage here results in lethality. The MAT/HML/HMR loci are not identical and perhaps sequence differences in the locus are responsible. In any case, it would be worth eliminating this possibility.

This proposed additional experiment is similar to the suggestion from a previous reviewer to test lethality of MAT +ScHO in S. cerevisiae, and eliminates a trivial explanation for the striking difference in lethality for HO-generated cleavage at MAT and HML/HMR

We thank the reviewer for her/his comments.

Concerning the proposed experiment, we feel that the configuration of the MTLs in C. glabrata already addresses the question raised by the reviewer. Indeed, the three sexual loci HML, MAT and HMR, are almost strictly identical in sequence. We also wish to remind the reviewer that the main sequence divergence occurs between the alpha and a versions of the Y sequence, and thus, of the Ho recognition site itself, and we do not observe a difference in behaviour between MATa and MATalpha cells. Apart from location and the Y sequence, we can differentiate those loci only by:

- the presence of the Z2 and W sequences which are very short sequences in C. glabrata (7 bp and 18 bp respectively). HML and MAT share the Z2 sequence whereas MAT and HMR share W. If the presence of those sequences at MAT was the reason the reason why a Ho-DSB at MAT is lethal, then a Ho-DSB at HML and HMR should also be lethal and we know that this is not the case.

- 1 single SNP (at least for strain HM100) in the X region that differentiates HML from MAT and HMR (A (HML) 🡪 C (MAT and HMR)). If this SNP was the reason why a Ho-DSB at MAT is lethal, then a Ho-DSB at HMR should also be lethal and we know that it is not the case.

We thus know that sequence is not implicated in why an Ho-cleavage at MAT is lethal, while cleavage at HML or HMR is non-lethal as long as there is a template to repair with. Thus, we feel that the specific lethality truly depends on the presence/activity of the Ho protein at the MAT locus in *C. glabrata*.

As for the experiment in S. cerevisiae suggested by a previous reviewer, our reply had been about the sequence identity, as above. Furthermore, the absence or presence of lethality upon cutting by Ho, if the MTLs from *C. glabrata* were transferred into *S. cerevisiae*, could always be specific to that yeast, especially since we are using *S. cerevisiae*'s *HO* gene.

Finally, we have recently had a manuscript accepted in Yeast, that shows that cutting of MAT by S. cerevisiae's Ho does not kill cells from N. delphensis, a yeast species from the same clade as C. glabrata. We believe this reinforces the specificity of our observation, and have included this in the Discussion.

Reviewer #2: I am happy with the changes that have been made to the manuscript. The authors have addressed the point about residual lethality that I raised in my previous review.

The revised manuscript has been restructured and is presented in a clearer way than originally. The experiments and arguments are logical and show that:
ScHO endonuclease is lethal when expressed in C. glabrata.
ScHO cleaves all three Cg MTL loci (MAT, HML and HMR).
The lethality of ScHO is due specifically to it cutting the Cg MAT locus, no HML or HMR.
A CRISPR cut at Cg MAT is not lethal and is repaired by mating-type switching.

One small comment is that Figure 1 would be clearer if the HO sites were marked on the sequences, e.g. by underlining, because they are in different orientations for the two species. Also, please check the sequence shown for the Z1 region at Sc HMRa: it shows a 3-bp insertion compared to the Z1 sequences of Sc HMLalpha and MATalpha, and I think this is an error.

We agree with the reviewer that this part of the figure was not clear enough. The sequence written in Figure 1 is the Ho site without any extra bp (24bp in S. cerevisiae and 26 bp in C. glabrata). We have clarified this information in the legend of this figure by adding the following sentence: “The Ho site sequence of each locus and species is shown in yellow.”.

In Figure 5, I am not sure that inclusion of the experiments with additional CRISPR guide RNAs (Ya1, Ya3, Ya4) has added anything to the manuscript and I would suggest dropping these.

We thank the reviewer for her/his comments and agree with her/him. The results with the additional CRISPR guide RNAs (Ya1, Ya3, Ya4) have been removed from the manuscript.

Reviewer #3: Many, though not all, of the reviewers' requests have been addressed. The most useful are the addition of data in Table 2 indicating that HML and HMR cutting is efficient, and the experiment in Fig. 6 addressing possible lethality caused by cutting at sites other than MAT. The introduction is also much improved. However, i don't think that adding extra gRNAs (Figure 5) has helped. Only one gRNA (the one described in the original submission) induced cutting. Looking at Fig. 5, this is not surprising. For Ya1 and Ya3, the cut site is some distance from the guide, and there is a lot of evidence that distance reduces efficiency. For Ya4, the PAM site is a very inefficient NAG, and this is part of the gRNA sequence. I don't think Cas9 will cut within a guide sequence. I understand that the authors were trying to address a reviewer's comment, but the added experiments add more confusion rather than clarifying. I suggest reverting to the original.

We thank the reviewer for her/his comments and agree with her/him. The results with the additional CRISPR guide RNAs (Ya1, Ya3, Ya4) have been removed from the manuscript to revert to the original.

Reviewer #4: The revised manuscript ‘A single HO-induced double-strand break at the MAT locus is lethal in Candida glabrata’ has adequately addressed the most of the considerations I posed during its initial review. The added discussion regarding the evolution and mechanism of mating type switching is welcome, as is the inclusion of the experimental questions in the results section.

There are three outstanding concerns with the revised manuscript.

1) It is presumed that the C. glabrata HO endonuclease is functional, yet doesn’t result in lethality when expressed, but there is 58% identity (82% similarity) with the S. cerevisiae homolog. Has there been biochemical analysis of its cutting efficiency already reported? If not, an EMSA experiment, could demonstrate whether both endonucleases have the capacity to cut at the same efficiency.

Previous experiments published by our lab (Boisnard et al., 2015) showed that in our expression system, C. glabrata’s Ho failed to induce mating-type switching in S. cerevisiae and induced mating-type switching in C. glabrata very rarely.

We assume throughout our work that mating-type switching/lethality reflects efficient cutting, since these events are caused by the expression of an endonuclease, as expected from all such experiments in many species. This thus suggests that C. glabrata’s Ho endonuclease is poorly efficient (contrary to S. cerevisiae’s Ho) and this is perhaps why it does not induce any lethality. However, we know that a highly efficient cut at MAT *per se* is not responsible for lethality, since Cas9 expression induces mating-type switching at a high level (Table 3), without lethality.

As for the EMSA experiment suggested, several problems arise: binding experiments of endonucleases on their DNA substrate are more difficult than such experiments for other DNA-binding proteins, such as transcription factors, since the substrate is cut. Even so, a gel-shift experiments would demonstrate the capacity to bind, not to cut. In order to measure cutting, we would need to have an in vitro assay for the endonuclease activity. No biochemical analysis of C. glabrata’s Ho has been reported to our knowledge. Furthermore, whereas people have been working with S. cerevisiae’s Ho endonuclease for decades, few biochemical analyses have been published, and results have not been reproduced (Kostriken et al., Cell 35, 167-174, 1983; Shibata et al., The Journal of Biological Chemistry 259:16,10499-10506, 1984; Jin et al., The Journal of Biological Chemistry 272:11, 7352-7359, 1997). To our knowledge, no one has succeeded in over-expressing Ho for purification in bacteria or yeast, nor in working properly with yeast-cell lysates with an Ho activity.

2) The low lethality when CAS9 is expressed – could this also be due to low expression or inefficient cutting?

Clearly it is capable of cutting, otherwise you wouldn’t see any switching, but how can one distinguish between low efficiency and strong selection for escaping the cleavage of MAT (such as by mutating the plasmid as the authors suggest)?

Yes, as stated above, in this work, efficient switching reflects efficient cutting. Thus, we suppose Cas9 to be sufficiently expressed to induce cutting at the level needed to iduce the massive switching observed.

We did observe what we assumed to be selection for escaping Cas-9 cleavage in liquid medium, because of the presence of a higher percentage of non-switched cells, than on plates. If such selection was very strong, it would not escape our notice on plates, where individual colonies are examined and subcloned. Such selection would lead to a high level of non-switched colonies. Furthermore, if escaping the cut was easily achievable by mutating the expression plasmid, we would expect this to also happen with the Ho expression plasmid since it has the same backbone, while in Ho liquid induction experiments, unswitched cells represent only 10% of the total.

Thus, again, we believe the results presented show that lethality is caused by Ho interacting with the MAT locus in C. glabrata.

3) I still find presentation of the figures to be problematic with the text. For example, in Figure 2 there are two strain that are initially discussed (HM100 and HM100 Δrad51; lines 178-182) and the other four strains that are not discussed until after Figure 3 has been presented (lines 222-232).

We have modified figures in such way that the reader does not have to go back and forth with the figures while reading the manuscript. The two first paragraphs refer now only to Figure 2. The third and fourth paragraphs refer to Figure 3. The fifth paragraph refers to Figure 4 etc…

Likewise, the text regarding data presented in Figure requires us to go back to Figure 3 to have a control to compare it. For experts in the field, this may not be an issue, but for those new to the field or still in their scientific training, this is very confusing and can be easily rectified. For example, in the latter case, adding the data from strain SL09 to Figure 6 with a note in the legend that it is the same data as in Figure 3 would suffice and would allow for the statistical significance to be conveyed on the graph in addition to in the text.

We agree with the reviewer that the reading of the figures is difficult to follow for people who are not experts in the field. As suggested, we have added data already presented in previous figures in Figure 5 and 6 to allow for the statistical significance to be conveyed on the graph in addition to in the text.

Minor notes:

Line 161 is written in the present tense, yet the rest of the section is written in the past tense.

We thank the reviewer for having noticed this. We have changed the present tense for the past tense.

Line 344: ‘inducting’ should be inducing?

We thank the reviewer for having noticed this. It has been corrected.

Line 363: what is meant by ‘some sexual identity of cells in maintained in C. glabrata’?

We meant that sexual identity is maintained in some C. glabrata cells, since previous work has shown that MATa cells only express the “a” information. However, this is less clear in MATalpha cells, where both “alpha” and “a” informations can be detected in the same cell since the HMRa locus is transcriptionnally "leaky". We have modified the sentence to:

" Since it has been shown that sexual identity of cells is maintained in C. glabrata, at least in MATa cells (i.e., MATa cells express MATa identity, but MATalpha cells have no mating-type specific expression of key genes, 21,22), we asked whether this “donor preference” from S. cerevisiae is conserved in C. glabrata at the MAT locus, and also whether HML and HMR use a preferential template for repair."

We hope this satisfies the reviewer.

**Changes made in the manuscript**

- Figure 2, 3, 5 and 6 as well as their legend have been changed.

- S2 Fig’s name has been changed for “S2 Fig. Mating-type screened by PCR at *MAT* in different strains.”.

- S2 Table has been modified to integrate strains SL0A and SL0B

- We add sub-sections in Table 2 for more clarity in our manuscript.

- First sub-section in Table 1 has been moved in the table.

- One correction has been made in the paragraph “*All three sites are cut by Ho in C. glabrata, including the one at HMR*” of the results section, accordingly the note of reviewer 4.

- Paragraph “*The lethality induced by the DSB at* MAT *is specific to* Sc*Ho*“ of the Results section have been rewritten and Table 3 has been modified to take off the Cas9 results obtained with gRNA Ya1, Ya3 and Ya4 and accordingly to the note of reviewer 4.

- Since results first contained in Figure 3 have been moved to Figure 2, citations of Figures in the paragraph “*Cleavage at HML and HMR is not an important contributor to lethality”* of the Results section have been changed.

- Since our paper on mating-type switching in N. delphensis was very recently accepted in Yeast, we have added it to the Discussion section and corrected some odd sentences in this section.

**Have all data underlying the figures and results presented in the manuscript been provided?**
Large-scale datasets should be made available via a public repository as described in the *PLOS Genetics* [data availability policy](http://journals.plos.org/plosgenetics/s/data-availability), and numerical data that underlies graphs or summary statistics should be provided in spreadsheet form as supporting information.

Reviewer #1: Yes

Reviewer #2: Yes

Reviewer #3: Yes

Reviewer #4: Yes

PLOS authors have the option to publish the peer review history of their article ([what does this mean?](https://journals.plos.org/plosgenetics/s/editorial-and-peer-review-process#loc-peer-review-history)). If published, this will include your full peer review and any attached files.


If you choose “no”, your identity will remain anonymous but your review may still be made public.


**Do you want your identity to be public for this peer review?** For information about this choice, including consent withdrawal, please see our [Privacy Policy](https://www.plos.org/privacy-policy).

Reviewer #1: No

Reviewer #2: No

Reviewer #3: No

Reviewer #4: No
